# Correction: McLoughlin, E.C.; O’Boyle, N.M. Colchicine-Binding Site Inhibitors from Chemistry to Clinic: A Review. *Pharmaceuticals* 2020, *13*, 8

**DOI:** 10.3390/ph13040072

**Published:** 2020-04-20

**Authors:** Eavan C. McLoughlin, Niamh M. O’Boyle

**Affiliations:** School of Pharmacy and Pharmaceutical Sciences, Trinity Biomedical Sciences Institute, Trinity College Dublin, Dublin D02, Ireland; niamh.oboyle@tcd.ie

We, the authors, wish to make the following corrections to our paper [1]: 

The compound with the code name BAL27862 is Avanbulin, not Plinabulin.

This affects:

Table 1 (page 25) – the entry for Plinabulin should not have BAL27862 after it. This entry refers to Plinabulin (BPI-2358) in clinical trials. The structural features listed alongside this entry are incorrect (refer to **75**, avanbulin). The structural features of Plinabulin include a piperazinedione structure. 

The structure of Plinabulin not currently included in the text. The structure is shown below for reference:


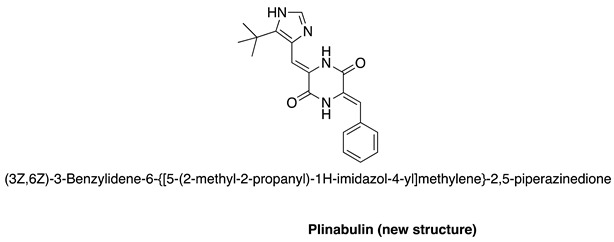


Table 1 (page 25) – entry for Lisavanbulin should state “Lysine prodrug of Avanbulin (**75**)” in column 3 (structural features).

Page 26 – Section 5.6 refers to Avanbulin (BAL27862) (**75**).

The last line of Section 5.6 should be removed (“It is also known as plinabulin and is now in clinical trials.”) It should read: “It is also known as avanbulin.”

Page 32 – Section 6.2.2. 

This paragraph refers to Beyond Spring’s compound, Plinabulin (provisional name **BPI-2358**, formerly **NPI-2358**). References to BAL27862 and **75** should be removed from this paragraph (first line).

Page 33 – Section 6.2.3. 

The first line should read “... prodrug of BAL27862/avanbulin (**75**, Figure 11) ...” (i.e., plinabulin changed to avanbulin).

The authors would like to apologize for any inconvenience caused to the readers by these changes.
